# Immune-related adverse events associated with the use of immunotherapy in patients with B-cell lymphoblastic leukemia: A protocol for a systematic review and meta-analysis

**DOI:** 10.1097/MD.0000000000032987

**Published:** 2023-03-24

**Authors:** Bianca K. Kanovengi, Bongani B. Nkambule, Edwig Hauwanga, Tawanda M. Nyambuya

**Affiliations:** a Department of Health Sciences, Faculty of Health and Applied Sciences, Namibia University of Science and Technology, Windhoek, Namibia; b School of Laboratory Medicine and Medical Sciences, College of Health Sciences, University of KwaZulu-Natal, Durban, South Africa; c School of Pathology, Department of Molecular Medicine and Haematology, Faculty of Health Sciences, University of the Witwatersrand, Johannesburg, South Africa.

**Keywords:** acute lymphoblastic leukemia, adverse events, B-cells, immunotherapy

## Abstract

B-cell acute lymphoblastic leukemia (B-ALL) is a neoplasm of immature B-cells that is more prevalent in children. Despite successful remission rates in patients with B-ALL on chemotherapy, the risk of relapse is high. This has paved way for highly active immune and cell therapies to be intensively explored. However, the efficacy and immune-related adverse events (AE) associated with the use of immunotherapies remain elusive.

**Methods:**

This protocol has been prepared in accordance with the Preferred Reporting Items for Systematic Review and Meta-Analysis Protocols 2015 guidelines. Cochrane Central Register of Controlled Trials, MEDLINE, and Embase electronic databases will be searched to retrieve relevant interventional studies. Two reviewers (BKK and EH) will autonomously search and identify relevant studies using a preset inclusion and exclusion criteria. A predefined data extraction sheet will be used to extract relevant data items. The risk of bias will be assessed by 2 reviewers (BKK and BBN) using the Cochrane risk-of-bias tool for randomized controlled trials and the Downs and Black Checklist for nonrandomized controlled trials. A third reviewer (TMN) will be consulted for any discrepancies. The Grading of Recommendations Assessment Development and Evaluation will be used to assess the strengths of evidence by 2 reviewers (BBK and TMN). The *I*² and Chi-squared statistical tests will be used to investigate statistical heterogeneity across studies. An *I*² value of > 50% will be considered substantial heterogeneity and a random-effects model will be used. Data analysis will be performed using Review Manager (RevMan V.5.3) statistical software.

## 1. Introduction

The global prevalence of acute lymphoblastic leukemia (ALL) has increased over the years and is the most frequently diagnosed leukemia in low to middle income countries.^[1,2]^ In the subSaharan African region, hematological malignancies are ascribed as one of the major causes of morbidity and mortality.^[2]^ In children and young adults, ALL is the common diagnosed cancer,^[3]^ whilst in the adult population, it is the second prevalent cancer.^[4]^ Although the exact etiology of ALL remains elusive, environmental and genetic factors such as ironizing radiation, previous chemotherapy or radiotherapy, and gene mutations, respectively, have been implicated.^[5]^ It is notable that most of ALL cases are due to the clonal proliferation of abnormal precursor B cells.^[4]^

B-cell acute lymphoblastic leukemia (B-ALL) is a malignancy that is committed to the B-cell lineage and is characterized by limited differentiation, uncontrollable cell proliferation and survival.^[6]^ As a result, standard treatment strategies such as chemotherapy and radiotherapy used in patients with B-ALL aims to antagonize tumorigenesis and progression.^[7]^ Despite the notable treatment efficacy of chemotherapy in B-ALL cases, a staggering 40% to 50% of patients relapse.^[8–10]^ Even worse, patients with relapse or refractory (R/R) ALL have bad prognosis, notwithstanding the use of aggressive treatment.^[11]^ Consequently, this has attracted attention to the exploration of alternative therapeutic approaches such as risk-targeted therapy and immunotherapy.^[12]^ Over the past years, immunotherapy has notably revolutionized the treatment of B-ALL and continues to improve the clinical outcomes of R/R B-ALL cases.^[11,13]^ The development of monoclonal antibodies, bispecific (BiTE) antibody constructs, and chimeric antigen receptor (CAR) T-cell therapies has significantly improved the prognosis of B-ALL and are inducing a paradigm shift in the treatment strategies.^[14–16]^

The use of Blinatumomab and Tisagenlecleucel, a BiTE antibody and a CD19 CAR T-cell, respectively in patients with B-ALL is associated with high rates of complete remission (CR), event free survival, overall survival (OS), minimal residual disease and progression free survival (PFS), even in cases with high-risk features.^[17–20]^ However, the high incidence of adverse events (AEs) manifesting from the use of these immunotherapies present a major setback as far as their safety is concerned. The severity of the AEs ranges from mild to life-threatening, and may be hematological, immunological, anatomical, or neurological in nature.^[21]^ Of major concern is the cytokine release syndrome (CRS), a systemic inflammatory response that is triggered by the induction of immunotherapy and presents with hypoxia, hypotension, and multiorgan dysfunction.^[22]^ The toxicity of CRS ranges from grade 1 to 4 depending on the severity of the symptoms and the intervention required thereof.^[23]^ Severe CRS, that is grade 3 or higher is a clinical menace as far as the degree of toxicity is concerned. The safety and efficacy of BiTE and CAR T-cells immunotherapies seems to be incomparable but debatable.^[24]^ Therefore, the systematic review and meta-analysis aims to investigate the efficacy of immunotherapy in patients with B-ALL and the immune-related AEs associated with its use.

### 1.1. Research question

1. What is the efficacy of immunotherapy in patients with B-ALL?
2. How common are the immune-related AEs associated with the use of different immunotherapies in B-ALL cases?

### 1.2. Objectives

1. To assess the efficacy of immunotherapy by determining the PFS, OS, MRD and CR rates in patients with B-ALL.
2. To determine the prevalence of severe CRS AEs following the administration of immunotherapy in B-ALL cases.

## 2. Methods protocol and registration

This protocol was prepared according to the Preferred Reporting Items for Systematic Review and Meta-Analysis Protocols 2015 guidelines^[25]^ and is registered on PROSPERO, registration number: CRD42022343693.

### 2.1. Eligibility criteria

This systematic review and meta-analysis will include interventional studies (randomized and nonrandomized controlled trials) clearly reporting on immune-related AE and efficacy of immunotherapy in patients with B-ALL with no search restrictions.

### 2.2. Participants

Patients with B-ALL on immunotherapy irrespective of age and/or gender.

### 2.3. Intervention

This systematic review and meta-analysis will consider studies that have included patients who have received immunotherapy as monotherapy or in combination with chemotherapy.

### 2.4. Comparators

The comparators groups will be determined based on the various study designs. This will include patients receiving conventional chemotherapy or radiotherapy.

### 2.5. Outcomes

Primary outcome: Immune-related AEs

Secondary outcome: CR, event free survival, OS, MRD, or PFS.

### 2.6. Information sources

The following electronic databases will be searched for relevant studies: MEDLINE, EMBASE, and the Cochrane Central Register of Controlled Trials (CENTRAL) from inception to the January 21, 2023.

### 2.7. Search strategy

The search strategy will be set using medical subject headings terms and key words associated to the primary and secondary outcomes such as ALL, b cells, immunotherapy and the related synonyms. An example of a detailed MEDLINE search strategy without any language restrictions is shown in Table S1, Supplemental Digital Content, http://links.lww.com/MD/I487. Grey literature and the reference list of included studies will also be scanned for applicable studies.

### 2.8. Study records

#### 1.2.8. Data management.

Data will be extracted according to Preferred Reporting Items for Systematic Reviews and Meta-analyses criteria. The titles, abstracts, and full texts of selected studies will be used to assemble data applicable to the review. Two autonomous reviewers (BKK and EH). A third reviewer (TMN) will be consulted for mediation in the event of discrepancies.

### 2.9. Study selection

A standard predetermined data extraction form will be used to for data extraction. The following data will be extracted: author, year of publication, the country in which the study was conducted, type of immunotherapy, immune AE, and main findings reported. Data from enumerated studies will be documented using Microsoft Excel. The Mendeley (V.1.19.8) reference manager (Elsevier, Amsterdam, Netherlands) will be used for identification of possible duplicates.

### 2.10. Data items

The study population will be subdivided into patients who received CAR T cells and immune antibodies. Additional subdivision will be relapsed and nonrelapsed patients.

### 2.11. Risk of bias in individual studies

The risk of bias will be assessed by 2 autonomous reviewers (BKK and BBN) using the current Risk of Bias Cochrane tool for randomized as well as the Downs and Black checklist for nonrandomized controlled trials. A third reviewer (TMN) will be consulted for any disagreements.

### 2.12. Data synthesis

A summary table will be used to document and harmonize the main outcomes (immune AE and complete remission) of included studies. Statistical heterogeneity will be assessed utilizing the *I*² and chi squared where an *I*² value of 50% will be regarded as substantial heterogeneity. Below 50% heterogeneity will be deemed as moderate and below 25% as low. A subgroup analysis and meta-regression comparing the study estimates from different study-level characteristics, will be performed to find the origin of heterogeneity. Fisher exact test will be used to evaluate the association between the end points. A fixed effects model will be used to analyze data and a nonrandom-effects model will be used where the outcomes of the included studies are homogeneous. All data analysis will be performed using the Review Manager (RevMan V.5.3) software.

### 2.13. Confidence in cumulative evidence

The quality of evidence for all outcomes in included studies will be determined by 2 autonomous reviewers (BKK and BBN) using the Grading of Recommendations Assessment, Development and Evaluation tool.^[26]^ A third author (TMN) will be consulted for mediation in case of disagreements. The quality of evidence will be assessed using the Grading of Recommendations Assessment Development and Evaluation domains that include risk of bias, inconsistency, indirectness, imprecision, and publication bias.

## 3. Discussion

Although successful remission rates have been noted in patients with B-ALL on standard chemotherapy, the OS and disease-free resilience remain a challenge. As a result, the use of immunotherapies is being exploited as treatment options for R/R B-ALL. Despite significant improvement in disease outcomes, the use of immunotherapy in B-ALL cases is associated with outward effects, particularly immune-mediated AE. It remains to be determined whether the different types of immunotherapies, that is CAR T-cells and BiTEs have comparable efficacy rates and safety. The goal is to thus identify immunotherapy that is effective in treating B-ALL and tolerable. Therefore, this systematic review and meta-analysis aims to investigate the efficacy of immunotherapy in patients with B-ALL and the immune-related AEs associated with its use.

## Author contributions

**Conceptualization:** Bianca K. Kanovengi, Tawanda M. Nyambuya.

**Supervision:** Tawanda M. Nyambuya.

**Writing – original draft:** Bianca K. Kanovengi, Tawanda M. Nyambuya.

**Writing – review & editing:** Bianca K. Kanovengi, Bongani B. Nkambule, Edwig Hauwanga, Tawanda M. Nyambuya.

## Supplementary Material


