# Supplementary material for: Immune-related adverse events associated with the use of immunotherapy in patients with B-cell lymphoblastic leukemia: A protocol for a systematic review and meta-analysis
Source: Medicine (Baltimore). 2023 Mar 24;102(12):e32987. doi: 10.1097/MD.0000000000032987 (PMC10036004; doi:10.1097/MD.0000000000032987)
Supplement: Supplementary file 1 [file medi-102-e32987-s001.pdf]

Supplementary Table 1: PubMed search strategy

**Terms**	**Search terms**
**#1**	(acute lymphoblastic leukemia[MeSH Terms]) AND (b cells[MeSH Term]) = 1375
**#2**	((acute lymphoblastic leukemia[MeSH Terms]) AND (b cells[MeSH Terms])) AND Immunotherapy = 138
**Combined #1 and #2**	(acute lymphoblastic leukemia[MeSH Terms]) AND (b cells[MeSH Term]) ((acute lymphoblastic leukemia[MeSH Terms]) AND (b cells[MeSH Terms])) AND Immunotherapy = **108 hits**
